# Body Mass Index and Right Ventricular Structure: Insights From Observational and Mendelian Randomization Analyses

**DOI:** 10.1002/pul2.70103

**Published:** 2025-06-03

**Authors:** Julian Pott, Maximilian Kirchner, Jan K. Hennigs, Christoph R. Sinning, Hans Klose, Lars Harbaum

**Affiliations:** ^1^ Division of Respiratory Medicine, Department of Internal Medicine II University Medical Centre Hamburg‐Eppendorf Hamburg Germany; ^2^ Department of Cardiology, University Heart and Vascular Centre Hamburg, University Medical Centre Hamburg‐Eppendorf, Member of the German Centre for Cardiovascular Research (DZHK) Partner Site Hamburg/Kiel/Luebeck Hamburg Germany

**Keywords:** BMI, genetic prediction, imaging, right ventricle

## Abstract

Overweight and obesity have emerged as modifiable risk factors for right ventricular (RV) phenotypic changes, but their genetic relationship remains unclear. This study examined RV phenotypes using cardiac magnetic resonance imaging in European participants from the UK Biobank without overt heart disease. Observational and Mendelian randomization approaches, based on individual‐ and summary‐level genetic data, were integrated to assess the effects of BMI on RV imaging phenotypes. Among 33,801 individuals with a mean age of 64 years, 52% were women, 41% were overweight, and 18% were obese. Overweight and obese participants exhibited larger RV volumes and lower RV ejection fractions compared to normal‐weight participants, even after adjusting for left heart parameters, cardiometabolic risk factors, and diseases. One‐sample Mendelian randomization revealed that higher lifetime BMI was related to larger RV end‐diastolic volume (3.4 mL per standard deviation BMI increase, 95% CI 2.8–4.0 mL), RV end‐systolic volume (1.6 mL, 95% CI 1.3–1.9 mL), and stroke volume (1.8 mL, 95% CI 1.4–2.2 mL). Adjustment for left ventricular measures reduced these effect sizes by 51%–67%, but relationships remained statistically significant. Two‐sample Mendelian randomization confirmed these findings using robust methods and correction for pleiotropic outliers. While the observational associations were more pronounced in women than in men, the genetic effects were similar across sexes. In conclusion, the relationships between BMI and RV volumes were generally consistent across observational and genetic analyses. Genetic predisposition to higher lifetime BMI influenced RV volumes in a population with a low prevalence of cardiopulmonary diseases, an effect not fully explained by left ventricular measures. These findings suggest that managing overweight and obesity may help prevent structural RV remodeling.

Body mass index (BMI) is an established metric for assessing obesity‐related health risks and guiding weight management strategies. BMI is influenced by both environmental and genetic factors. The heritability of BMI is estimated to be 30%–40%, with common genetic variants accounting for more than half of this heritability, as demonstrated by large genome‐wide association studies [1]. BMI has been proposed as a modifiable risk factor associated with structural and functional changes in right ventricular (RV) phenotypes, which were found to be independent of left ventricular measures [2, 3, 4, 5]. These changes could contribute to RV dysfunction, particularly under conditions of chronic increased afterload, such as pulmonary hypertension, where the ability of the RV to adapt and preserve systolic function represents a major determinant of outcomes [6, 7]. Furthermore, BMI has been shown to influence treatment responses to pulmonary hypertension‐targeted drugs in clinical trials, and has been linked to survival in observational cohort studies of patients with pulmonary hypertension, although the direction remains unclear [8, 9, 10, 11].

Despite observational associations between BMI and RV imaging phenotypes, the genetic underpinnings of these relationships remain unclear. To address this, we hypothesized that genetically predicted BMI influences RV phenotypes in a manner consistent with their observational relationships. In this study, we integrated observational and genomic approaches to explore the association between BMI and RV imaging phenotypes. Using Mendelian randomization techniques, we analyzed individual‐ and summary‐level genetic data to evaluate the effects of genetically predicted BMI on clinically relevant RV imaging phenotypes derived from magnetic resonance imaging (e.g., volumes and ejection fraction). Mendelian randomization leverages the heritability of BMI to mitigate biases from environmental confounders and allow for causal inferences [12].

Observational analyses showed that higher BMI is associated with larger RV volumes and lower ejection fraction, independent of left ventricular measures, cardiometabolic risk factors, and diseases. Genetic analyses confirmed these independent associations for RV volumes but not ejection fraction. Our findings establish a genetic link between lifetime BMI and RV structure, specifically RV volumes, suggesting underlying mechanisms beyond the influence of the left ventricle (LV).

## 1 Methods

### 1.1 Cohort, Genotyping and Imaging

The UK Biobank is a multicentric cohort that recruited individuals aged 40 – 69 years in the UK from 2006 to 2010. It has collected information on health care data, biological samples, genotypes, and cardiac magnetic resonance imaging (MRI) data. Genotypes were called using two purpose‐designed arrays (UK BiLEVE Axiom and UK Biobank Axiom microarrays, Affymetrix, Santa Clara, CA, USA) and were imputed using the Haplotype Reference Consortium and merged UK10K and 1000 Genomes phase 3 reference panels. Biochemical analysis and genotyping were taken from participants at their initial baseline visit. Body weight and standing height were measured directly at the time imaging was performed. Cardiac MRI was performed on a sub‐cohort starting in 2016 during follow‐up visits at three assessment centers on a clinical wide bore 1.5 Tesla scanner (MAGNETOM Aera, Syngo Platform VD13A, Siemens Healthcare, Erlangen, Germany) [13]. Volumes of both ventricles across a cardiac cycle were captured from segmented images based on an extensively validated deep‐learning network model, which was trained on manual annotations (UK Biobank field IDs 24100 to 24109) [5, 14]. Imaging phenotypes included end‐diastolic volume (EDV in mL) and end‐systolic volume (ESV in mL), and derived stroke volume (SV in mL) and ejection fraction (EF in %) [5, 14]. For this study, we included participants with European ancestry, free from a history of myocardial infarction or heart failure, and available genetic and MRI data, who had not withdrawn consent as of June 2023. Details on quality control on genotype data and inclusion criteria for participants were reported previously for this data set of the UK Biobank imaging cohort [15].

### 1.2 BMI Groups and Observational Association

Participants were categorized into four BMI groups: underweight (BMI < 18.5 kg/m²), normal weight (BMI 18.5–24.9 kg/m²), overweight (BMI 25–29.9 kg/m²), and obese (BMI ≥ 30 kg/m²). Generalized linear regression models were used to assess the associations between BMI, either as ordinal or continuous variable, and RV imaging phenotypes. Results on BMI categories were expressed as least squares means and on continuous BMI as estimates with 95% confidence interval (CI) following standardization of BMI to a mean of 0 and standard deviation of 1. Covariates for adjustment were chosen based on known or possible associations with changes in RV phenotypes or weight status. Covariates included age, sex, height, height², imaging centre, triglyceride, low‐density lipoprotein cholesterol, high‐density lipoprotein cholesterol, glucose, glycated hemoglobin, history of diabetes, history of atrial fibrillation, history of arterial hypertension, and use of lipid‐lowering drugs. Height and height^2^ were chosen as covariates to ensure that results were indicative of weight status rather than overall body type, and to account for nonlinear relationships. Regression analyses were repeated with additional adjustment for corresponding LV measure to account for interventricular dependencies and to examine RV‐specific associations. We did not adjust for LV stroke volume due to its dependency on RV stroke volume in a closed loop system. In addition, the interaction term combining BMI and sex was included as a covariate, to assess the combined effect (interaction) of BMI and sex on the RV phenotypes.

### 1.3 Variant Selection and Instrument Variable

The instrument variable for BMI was constructed using variants (and their corresponding effect sizes) associated with BMI that achieved genome‐wide significance (*p* < 5 × 10⁻⁸) as reported by the GIANT Consortium (*N* = 322,154; European ancestry) [1]. Genetic variants that passed quality control in the UK Biobank participants included in the present study were clumped for linkage disequilibrium at *r* ^2^ < 0.1 within a ± 500 kb window. Clumping was performed within the UK Biobank imaging cohort rather than an external reference cohort. The weighted genetic instrument for each participant was calculated by summing the product of the effect sizes and the number of effect alleles across all selected variants. Variance explained (*r*²) and *F* statistics were obtained by regressing the genetic instrument on measured BMI. An analysis of variance was performed to test whether the additional proportion of variance in BMI explained by the genetic instrument, after accounting for basic covariates (age, sex, height, height^2^, centre, genotype array, and the first 10 genetic principal components), was significant. The null model, which included only the basic covariates, was compared against a model that also included the genetic instrument, providing sum of squares, *F*‐statistic, and *p*‐value, which were used to determine if the genetic instrument significantly improved the model fit.

### 1.4 One‐Sample Mendelian Randomization

We used two‐stage least‐squares regression models on individual‐level genetic data (also called one‐sample Mendelian randomization) to associate the instrument variable on BMI (exposure) with RV phenotypes (outcome) using the R‐package ivreg (version 0.6.2). This approach allowed us to directly compare Mendelian randomization and observational findings in a single population sample. In the first stage, the exposure is regressed on the genetic variants and covariates. In the second stage, the outcome is then regressed on the predicted values of the exposure from the first regression and the same covariates. Covariates included age, sex, height, height², genotype array, assessment centre, and the first 10 genetic principal components. Regression analyses were repeated with additional adjustment for corresponding LV measure and for the interaction term between sex and BMI. We did again not adjust for LV stroke volume, given its dependence on RV stroke volume. We included the covariates for both the endogenous variable and the instrumental variable. Results were expressed as estimates and 95% CI following standardization of the genetic instrument to a mean of 0 and standard deviation of 1. The Durbin–Wu‐Hausman test was used to assess endogeneity of the genetic variable, testing whether the estimates obtained from the genetic instrument are significantly different from those obtained from the observational analysis.

### 1.5 Two‐Sample Mendelian Randomization

We performed two‐sample Mendelian randomization using summary‐level association data provided by the GIANT Consortium (instrument variable) and previously published results from the UK Biobank imaging cohort on RV phenotypes [1, 15]. Results from UK Biobank imaging cohort, analyzed with alternative pipelines to capture imaging phenotypes and perform genome‐wide association testing, including sex‐stratified analyses, were also utilized [1, 15, 16, 17]. Effect estimates were primarily derived using the inverse variance‐weighted (IVW) method based on a multiplicative random‐effects meta‐analysis model as implemented in the TwoSampleMR R‐package (version 0.5.7) [5]. Additionally, we employed robust methods, including penalized MR‐Egger and weighted median, to further assess the validity of the genetic instruments [18]. To evaluate the presence of directional pleiotropy, we applied the MR‐Egger intercept test, which tests whether the genetic variants used as instrumental variables influence the outcome through pathways other than the exposure, and visually assessed funnel plots, where symmetry around the central effect estimate suggests the absence of systematic bias [19]. Furthermore, we applied MR pleiotropy residual sum and outlier (MR‐PRESSO) to detect any horizontal pleiotropic outlier [20]. To test for heterogeneity between sexes, we used Cochran's *Q* test for heterogeneity provided by the R‐package metaphor (version 4.2.0).

## 2 Results

**Figure 1:**
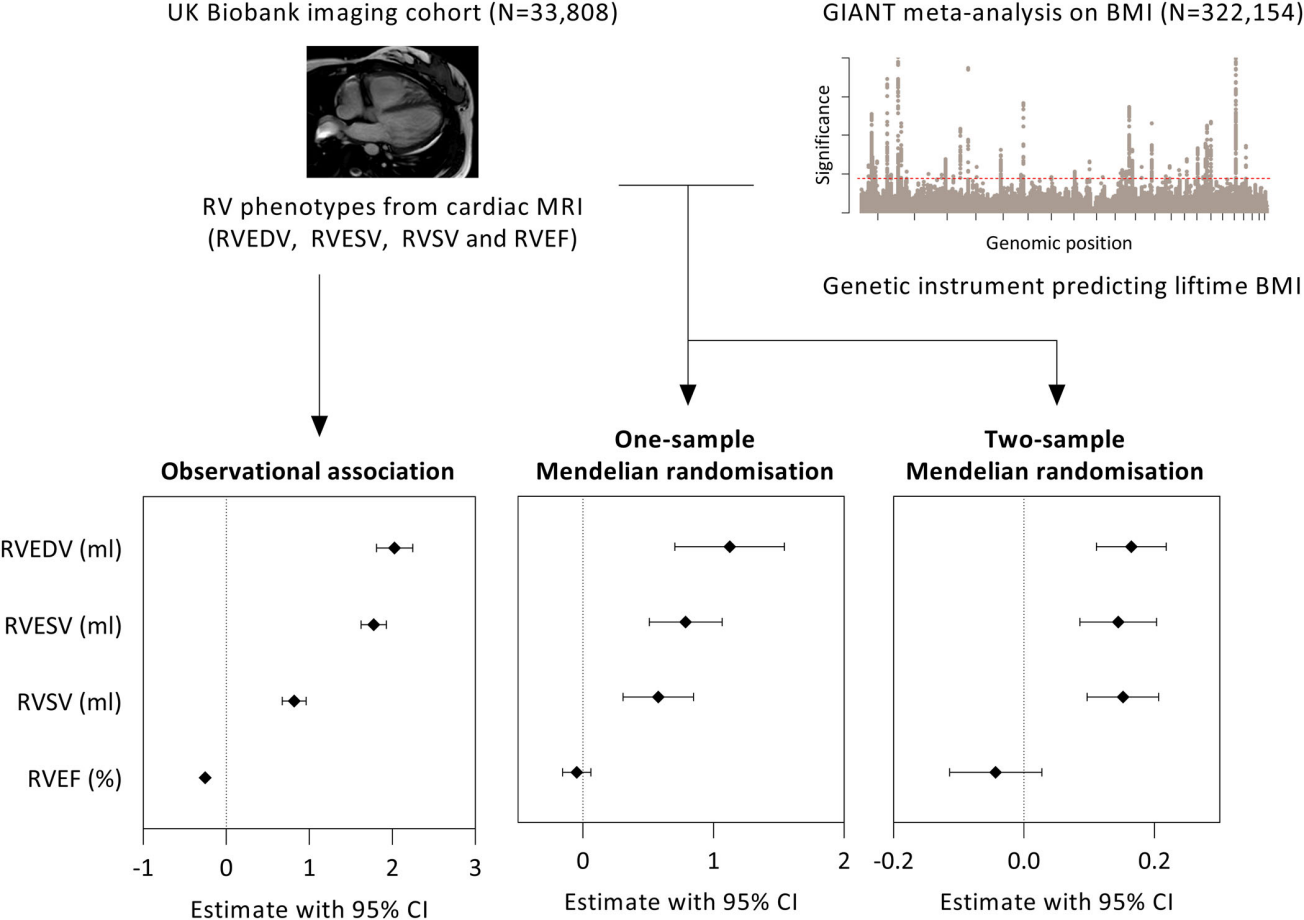
Consistency between observational associations and genetic predictions. Cardiac magnetic resonance imaging (MRI) was used to evaluate right ventricular (RV) volumes and function in 33,808 European participants from the UK Biobank imaging study. Genetic variants achieving genome‐wide significance (*p* < 5 × 10^−8^) in a meta‐analysis of 322,154 European individuals (GIANT consortium) were used to construct a genetic instrument for predicting body mass index (BMI). Both directly measured and genetically predicted BMI were analyzed in relation to RV imaging phenotypes, and effect estimates with 95% confidence intervals (CIs) were calculated. Observational and one‐sample Mendelian randomization models were adjusted for basic covariates and the corresponding left ventricular measures. Estimates correspond to the effect of a one standard deviation (SD) increase in BMI. RVEDV, RV end‐diastolic volume; RVEF, RV ejection fraction; RVESV, RV end‐systolic volume; RVSV, RV stroke volume.

We included 33,801 European participants from the UK Biobank imaging cohort, with a mean age of 64 years, of whom 52% were women. Overweight and obesity were common, with 41% and 18% of participants. Only 0.8% of participants were underweight, and they were grouped with those of normal weight for downstream analyses. Overweight and obese participants had higher rates of diabetes, hypertension, and use of lipid‐lowering drugs. Detailed characteristics of the cohort stratified by BMI groups are reported in Table 1. Distributions of measured RV volumes and ejection fraction are depicted in Supporting Information S1: Figure 1.

**Table 1:** Characteristics of participants from the UK Biobank imaging cohort.

Parameter	Unit	UK Biobank imaging cohort			
		Total	Normal weight	Overweight	Obese
			BMI < 25 kg/m^2^	BMI 25–29.9 kg/m^2^	BMI ≥ 30 kg/m^2^
		*N* = 33,805	*N* = 13,805	*N* = 13,922	*N* = 6,074
Age	Years	63.7 (7.5)	63.6 (7.6)	64 (7.5)	62.9 (7.4)
Female sex	%	52.2	61.2	44	50.5
Height	cm	169.3 (9.3)	168.7 (9)	170.1 (9.4)	168.8 (9.4)
Weight	kg	76.1 (15.1)	64.8 (8.8)	78.9 (9.6)	95.7 (13.7)
Waist circumference	cm	88.3 (12.7)	78.8 (8.3)	90.8 (8.1)	104 (10.6)
BMI	kg/m^2^	26.5 (4.4)	22.7 (1.7)	27.2 (1.4)	33.5 (3.5)
SBP	mmHg	138.2 (18.4)	134.4 (18.7)	140.1 (17.6)	142.6 (17.4)
DBP	mmHg	78.5 (10)	75.8 (9.8)	79.7 (9.6)	81.9 (9.5)
FEV_1_/FVC	—	0.7 (0.1)	0.7 (0.1)	0.7 (0.1)	0.8 (0.1)
LDL‐C	mmol/L	3.6 (0.8)	3.5 (0.8)	3.6 (0.8)	3.6 (0.9)
HDL‐C	mmol/L	1.5 (0.4)	1.6 (0.4)	1.4 (0.3)	1.3 (0.3)
Triglyceride	mmol/L	1.6 (1)	1.4 (0.8)	1.8 (1)	2 (1.1)
HbA1c	mmol/mol	34.9 (5)	34.3 (4.1)	35 (5)	36.2 (6.5)
Lipid lowering drug use	%	19.1	12.5	22.3	27.1
Diabetes mellitus (any)	%	3.5	1.3	3.6	8.4
Arterial hypertension	%	29	18.4	31.5	47.2
Atrial fibrillation	%	2.8	2.3	3	3.7
COPD	%	1.9	1.4	1.8	3.1

*Note:* Data are presented as arithmetic mean with standard deviation or as percentage.

Abbreviations: BMI, body mass index; COPD, chronic obstructive pulmonary disease; DBP, diastolic blood pressure; FEV_1_/FVC, forced expiratory volume in one second to forced vital capacity; HbA1C, glycated hemoglobin; HDL‐C, high density lipoprotein cholesterol; LDL‐C, low density lipoprotein cholesterol; SBP, systolic blood pressure.

### 2.1 Observational Relationships Between BMI and RV Imaging Phenotypes

Overweight and obese participants had larger RV volumes and lower RV ejection fraction (Figure 1). The adjusted least squares mean values across different BMI groups are shown in Table 2. Compared to participants with normal weight, overweight and obese participants had 5.1% and 11.6% larger RV end‐diastolic volumes, 5.6% and 12.1% larger end‐systolic volumes, 4.9% and 10.9% larger stroke volumes, and 0.4% and 0.9% lower ejection fraction. When further adjusting for LV measures, the associations between BMI and RV phenotypes were attenuated but remained statistically robust. Overweight and obese participants had a larger RV end‐diastolic volume by 0.8% and 2.9%, larger end‐systolic volume by 2.7% and 5.8%, and a reduced ejection fraction by 0.4% and 0.9%, respectively, compared to participants with normal weight and independent of LV measures. Thus, the smallest differences across BMI groups were observed for RV ejection fraction, but without attenuation following LV adjustment. When assessed as a continuous variable, increasing BMI was similarly associated with larger RV volumes and reduced ejection fraction following adjustments for LV measures (Table 3).

**Table 2:** Adjusted least square mean values of right ventricular (RV) imaging phenotypes within each of three bands of body mass index (BMI) in the UK Biobank cohort.

Phenotype	Model*	BMI groups in UK Biobank imaging cohort			*p* value for trend**
		Normal weight	Overweight	Obese	
		BMI < 25 kg/m^2^	BMI 25‐29.9 kg/m^2^	BMI ≥ 30 kg/m^2^	
		*N* = 13,805	N = 13,922	*N* = 6074	
RVEDV, mL	Basic	149.1	156.8	166.5	< 0.001
	Basic + LV	154.2	155.4	158.7	< 0.001
RVESV, mL	Basic	66.2	69.9	74.2	< 0.001
	Basic + LV	66.9	68.7	70.8	< 0.001
RVSV, mL	Basic	82.4	86.5	91.4	< 0.001
RVEF, %	Basic	56	55.8	55.5	< 0.001
	Basic + LV	56.6	56.4	56.1	< 0.001

*Note:* Data are presented as adjusted least square means.

Abbreviations: LV, left ventricle; RVEDV, RV end‐diastolic volume; RVEF, RV ejection fraction; RVESV, RV end‐systolic volume; RVSV, RV stroke volume.

* Basic model was adjusted for age, sex, height, height², imaging centre, triglyceride, low density lipoprotein cholesterol, high density lipoprotein cholesterol, glucose, glycated hemoglobin, history of diabetes, history of atrial fibrillation, history of arterial hypertension, and use of lipid‐lowering drugs. In a second model the corresponding LV measure was added as covariate.

** *p* values were generated from linear regression models with BMI bands treated as an ordinal variable.

**Table 3:** Direct comparison between observational associations and one‐sample Mendelian randomizations of body mass index (BMI) on right ventricular (RV) phenotypes in the UK Biobank imaging cohort.

Phenotypes	Model*	Observational association**			One‐sample Mendelian randomization**			Durbin‐Wu‐Hausman test
		Estimate	95% CI	*p* value	Estimate	95% CI	*p* value	*p* value
RVEDV, mL	Basic	7.6	7.2 to 7.9	< 0.001	3.4	2.8 to 4	< 0.001	< 0.001
	Basic + LV	2	1.8 to 2.2	< 0.001	1.1	0.7 to 1.5	< 0.001	< 0.001
RVESV, mL	Basic	3.5	3.3 to 3.7	< 0.001	1.6	1.3 to 1.9	< 0.001	< 0.001
	Basic + LV	1.8	1.6 to 1.9	< 0.001	0.8	0.5 to 1.1	< 0.001	< 0.001
RVSV, mL	Basic	4	3.8 to 4.2	< 0.001	1.8	1.4 to 2.2	< 0.001	< 0.001
RVEF, mL	Basic	−0.2	−0.3 to −0.2	< 0.001	−0.1	−0.2 to 0	0.14	0.47
	Basic + LV	−0.3	−0.3 to −0.2	< 0.001	0	−0.2 to 0.1	0.37	0.97

Abbreviations: CI, confidence interval; LV, left ventricle; RVEDV, RV end‐diastolic volume; RVEF, RV ejection fraction; RVESV, RV end‐systolic volume; RVSV, RV stroke volume.

* Basic adjustment for observational association included age, sex, height, height², imaging centre, triglyceride, low‐density lipoprotein cholesterol, high‐density lipoprotein cholesterol, glucose, glycated hemoglobin, history of diabetes, history of atrial fibrillation, history of arterial hypertension, and use of lipid‐lowering drugs. Basic adjustment for genetic prediction included age, sex, height, height², genotype array, assessment centre, and the first 10 genetic principal components. In second models the corresponding LV measure was added as covariate.

** Effect estimates and 95% CI corresponded to an increase of one standard deviation in directly measured or genetically predicted BMI.

We next tested whether the effect of BMI on RV phenotypes was sex‐specific. The interaction between BMI and sex was significant for RV end‐diastolic and stroke volume, both for BMI as categorized and continuous variable, suggesting that sex modifies the effect of BMI on these two RV phenotypes (Supporting Information S1: Table 1). The increase in RV end‐diastolic and stroke volume in overweight and obese participants were larger in women compared to men. Based on adjusted least squares mean values across different BMI groups, overweight and obese women had 6.2% and 14.9% larger RV end‐diastolic volumes, and 6.1% and 14.3% larger stroke volumes, compared to women with normal weight. While differences were attenuated in men with 4.2% and 8.7% larger RV end‐diastolic volumes and a 3.9% and 8.3% large stroke volume for overweight and obese man, compared to men with normal weight. There was no significant interaction between BMI and sex for RV end‐systolic and ejection fraction, indicating a consistent effect of BMI across sexes (Supporting Information S1: Table 2).

### 2.2 Direct Comparison of Observational and Genetically Predicted Effects of BMI on RV Imaging Phenotype Using One‐Sample Mendelian Randomization

To enable direct comparisons between observational and genetically predicted effects, we first employed a one‐sample Mendelian randomization approach using a stringent genome‐wide significance threshold to select genetic variants as instrumental variables for BMI. The final genetic instrument included 98 uncorrelated variants, which were strongly associated with BMI in the UK Biobank imaging cohort (*F*‐statistic of 414; Supporting Information S1: Table 3). Including this genetic instrument in a model adjusted for age, sex, and height significantly reduced the residual variance in BMI, as indicated by an *F*‐statistic of 423 (*p* < 0.001). In this model, the genetic instrument explained 1.2% of the variance in measured BMI (Supporting Information S1: Figure 2).

Genetic predisposition to higher lifetime BMI was significantly related to larger RV end‐diastolic volume, larger end‐systolic volume, and larger stroke volume. No significant relationship was found with RV ejection fraction (Table 3). To determine whether the predicted effects of BMI on RV volumes were independent of their effects on LV volumes, we repeated the analyses with additional adjustment for the corresponding LV measure. Genetically predicted BMI, adjusted for LV measures, was linked to larger RV end‐diastolic volume and larger end‐systolic volume. Thus, effect estimates between BMI and RV volumes remained statistically significant, but effect sizes decreased by 67% for end‐diastolic volume and 51% for end‐systolic volume (Table 3).

Comparing the genetically predicted and observational associations, the findings were generally consistent for RV volume measures. Effect estimates were approximately half of those from observational associations and observational reduction in RV ejection fraction with increasing BMI was not supported by the genetic analysis (Table 3 and Figure 1). No significant interaction between BMI and sex was observed in the one‐sample Mendelian randomization models, indicating that genetically predicted BMI had similar effects on RV phenotypes in both women and men (Supporting Information S1: Table 4).

### 2.3 Identification of Genetically Predicted Effects of BMI on RV Imaging Phenotype Using Two‐Sample Mendelian Randomization

To account for the limitations of one‐sample Mendelian randomization, which can be more susceptible to weak instrument bias and overfitting, we used summary‐level data to perform two‐sample Mendelian randomization. We used the identical set of variants associated with BMI at genome‐wide significance threshold for the instrument variable (Supporting Information S1: Table 3).

We confirmed significant relationships for RV end‐diastolic volume, end‐systolic volume, and stroke volume, with concordant directions of effect, suggesting a possible causal interaction (Figure 1). Again, no significant effect was found for RV ejection fraction. Robust methods, including MR‐Egger and weighted median estimators, were used to account for potential violations of the exclusion restriction assumption, where instrumental variables may influence the RV through pathways other than BMI. These methods supported the results, showing concordant directions of effect, although with wider confidence intervals (Table 4). Based on MR‐Egger regression intercept and funnel plots, no evidence of directional horizontal pleiotropy was detected (Supporting Information S1: Table 5 and Figure 3). We identified a single potential horizontal pleiotropic outlier in the analysis of RVEDV and RVESV, but results remained similar after its exclusion (Supporting Information S1: Table 6 and Figure 3).

**Table 4:** Two‐sample Mendelian randomizations to predict the effect of body mass index (BMI) on right ventricular (RV) phenotypes in the UK Biobank imaging cohort.

Two‐sample Mendelian randomization					
Phenotypes	Model		Estimate	95% CI	*p* value
RVEDV	Primary	IVW	0.16	0.11 to 0.22	< 0.001
	Sensitivity	MR Egger	0.18	0.04 to 0.32	0.016
		WM	0.13	0.05 to 0.2	< 0.001
RVESV	Primary	IVW	0.14	0.09 to 0.2	< 0.001
	Sensitivity	MR Egger	0.19	0.04 to 0.35	0.017
		WM	0.16	0.09 to 0.24	< 0.001
RVSV	Primary	IVW	0.15	0.1 to 0.21	< 0.001
	Sensitivity	MR Egger	0.13	−0.02 to 0.27	0.083
		WM	0.09	0.01 to 0.17	0.034
RVEF	Primary	IVW	−0.04	−0.11 to 0.03	0.23
	Sensitivity	WM	−0.11	−0.2 to −0.02	0.019
		MR Egger	−0.12	−0.31 to 0.06	0.19

Abbreviations: CI, confidence interval; IVW, inverse variance‐weighted; LV, left ventricle; RVEDV, RV end‐diastolic volume; RVEF, RV ejection fraction; RVESV, RV end‐systolic volume; RVSV, RV stroke volume; WM, weighted median.

Results were also consistent across different sets of summary‐level data from genome‐wide association studies on the UK Biobank imaging cohort, which were generated using different analytical pipelines, and there was no evidence for heterogeneity between sexes in these data (Supporting Information S1: Figure 4 and Table 7).

## 3 Discussion

Understanding the clinical factors contributing to the heterogeneity in RV adaptation and structural remodeling is crucial for developing effective treatment strategies. This study provides both observational and genetic evidence of a relationship between BMI and RV volumes, with genetically predicted higher lifetime BMI being associated with larger RV end‐diastolic, end‐systolic, and stroke volume. Observational and genetic estimates were directionally consistent and remained robust after adjusting for LV measures, suggesting that weight management strategies may directly influence the RV structure.

Our observational associations in participant from the UK Biobank align with previous MRI‐based findings. In the MESA study, overweight and obese participants without overt heart disease had larger RV volumes, including stroke volume, compared to participants with normal weight, while RV ejection fraction was slightly reduced [2]. The increased RV volumes could partly be explained by the increased blood volume associated with greater body mass, as reflected by the higher stroke volume and consecutively, higher cardiac output in overweight and obese individuals [2, 21]. However, results from both MRI studies (MESA and UK Biobank) were adjusted for height and were only partially attenuated by adjustment for corresponding LV measures, suggesting that factors related to body mass, beyond just blood volume, likely contribute to structural RV changes. Both studies observed a slight reduction in RV ejection fraction. While cardiac MRI may offer more accurate measurements, these observations are consistent with a report showing an association between increasing BMI and decreasing echocardiographic RV strain in overweight and obese individuals free from pre‐existing heart diseases [4]. While a more recent study from a large referral centre, observed a positive correlation between BMI and echocardiographic tricuspid annular plane systolic excursion, observational associations remain conflicting, and differences in imaging techniques and cohorts may explain some of the controversies [3].

Since BMI is influenced by genetic factors, we employed genetic variants as proxies for BMI to statistically infer causal relationships through Mendelian randomization [1]. Leveraging this genetic framework, we identified a genetic relationship between increased lifetime BMI and larger RV volumes. Although previous genetic evidence has demonstrated a link between BMI and changes in LV morphology, our findings indicate that the effect of BMI on RV volumes, while attenuated after adjustment for LV measures, remained statistically significant [22, 23]. These findings highlight the potential for weight management strategies to mitigate RV remodeling in patients with higher BMI at risk for RV dysfunction. In addition to reducing blood volume, which directly alleviates hemodynamic congestion and cardiac workload, targeting weight loss could reduce epicardial adipose tissue, relieving pericardial restraint, systemic inflammation, insulin resistance, hormonal activation, adipokines including leptin, dyslipidaemia, and other obesity‐related factors that may contribute to impaired cardiac structure and function [24, 25, 26, 27, 28].

We found a stronger observational effect of BMI on RV volumes in women, but did not observe a significant interaction between BMI and sex for RV ejection fraction. Mendelian randomization did not support sex‐specific genetic effects of BMI, implying that environmental and lifestyle factors may play a relevant role in driving the observed sex differences in the relationship between BMI and RV phenotypes. However, by using a sex‐combined cohort to develop the genetic instrument for BMI, the instrument may dilute sex‐specific genetic differences [1]. Thus, our findings highlight that further research into sex‐specific mechanisms of the relationships between BMI and RV phenotypes is required.

We acknowledge limitations in our study. By focusing on individuals of European descent, the generalizability of our findings is limited in non‐European populations. Both the observational and genetic analyses were cross‐sectional, preventing us from establishing the timing and progression of changes in BMI and RV phenotypes. Biochemical analyses were performed at baseline and were potentially confounded by factors that may have occurred between baseline and imaging visit. While the large cohort size preserves statistical significance after adjusting for LV parameters, the observed attenuation in the relationship between BMI and RV measures is important, suggesting that a sizeable portion of the influence of BMI may be biventricular. We cannot entirely rule out the possibility that the residual BMI effect on the RV may be influenced by measurement limitations associated with the thin‐walled RV or from unrecognized comorbid pathology. In addition, observational studies are inherently susceptible to residual confounding, and the influence of unmeasured variables contributing to the relationship between BMI and RV phenotypes may persist despite model adjustments. This is particularly important for the association between BMI and RVEF, where genetic relationships do not support the observational data. Although Mendelian randomization provides causal insights, whereas cross‐sectional observational data does not, which limits the ability to infer causality directly from the observed associations between BMI and RV phenotypes. In this regard, clinical studies are warranted to establish the effects of BMI modulation on RV parameters, such as during treatment with agonists for the glucagon‐like peptide‐1 (GLP‐1) and glucose‐dependent insulinotropic polypeptide (GIP) receptors, which have been shown to lower BMI in overweight and obese individuals [29, 30].

While Mendelian randomization is a powerful tool, key assumptions, such as the independence of genetic variants from confounders and the exclusion restriction, cannot be fully tested. Using the same cohort for both observational and Mendelian randomization analyses may potentially inflate some of these biases, including shared confounding, overestimation of genetic associations, and pleiotropy. To address these concerns, we employed multiple sensitivity analyses within our two‐sample Mendelian randomization approach, accounting for potentially pleiotropic variants and applying robust methods. However, we cannot completely rule out the possibility of residual horizontal pleiotropy, where genetic variants affect the outcome through pathways other than the exposure. Furthermore, the variance in BMI explained by the genetic instrument in this cohort was relatively modest (1.2%), which may have led to an underestimation or bias in the estimated effects.

Although our study aligns observational and genetic data, it lacks mechanistic insights that could pinpoint specific molecular targets underlying the potential causal relationship. This limitation highlights the need for further basic science investigations to uncover the biological pathways driving the associations.

In summary, our study strengthens the evidence for an interaction between overweight/obesity and structural changes in the RV. While increased BMI seems to be genetically related to increased RV volumes, its observed impact on RV systolic function appears to be mediated by other factors. Further studies are needed that incorporate advanced imaging techniques or pressure‐volume analysis, and that integrate molecular phenotypes.

## Author Contributions

Study concept, statistical analysis: Lars Harbaum. Manuscript draft: Julian Pott and Lars Harbaum. Manuscript review and interpretation of results: Julian Pott, Maximilian Kirchner, Jan K. Hennigs, Christoph R. Sinning, Hans Klose and Lars Harbaum. All authors have approved the final version of the manuscript.

## Ethics Statement

This study was covered by the general ethical approval for UK Biobank studies from the Northwest Research Ethics Committee under reference number 11/NW/0382.

## Conflicts of Interest

All authors have approved the current version of the manuscript before submission and declared that the submitted work is original and has not been published elsewhere (nor is it under consideration for publication) in any language, including English. No financial conflict of interest has arisen from any author that interferes with the study design, methods, or results.

## Supporting information

Supporting Information

## Data Availability

The summary data that support the findings of this study are available from the corresponding author upon reasonable request. UK Biobank data can be accessed via https://www.ukbiobank.ac.uk. Data from the GIANT consortium can be accessed via https://portals.broadinstitute.org/collaboration/giant/index.php/GIANT_consortium.
