# Supplementary material for: Body Mass Index and Right Ventricular Structure: Insights From Observational and Mendelian Randomization Analyses
Source: Pulm Circ. 2025 Jun 3;15(2):e70103. doi: 10.1002/pul2.70103 (PMC12130632; doi:10.1002/pul2.70103)
Supplement: Supplementary file 1 — Supporting Information [file PUL2-15-e70103-s001.docx]

SUPPLEMENT

Supplementary tables 1 to 7

Supplementary figure 1 to 4

**Supplementary table 1** – Results of the interaction term between body mass index (BMI) and sex from multivariable regression models assessing the effect of BMI on right ventricular (RV) imaging phenotypes.

| RV phenotype | Unit | Model* | Estimate | 95% confidence interval | | p-value |
| --- | --- | --- | --- | --- | --- | --- |
|  |  |  |  | Lower | Upper |  |
| BMI as ordinal variable | | | | | | |
| RVEDV | ml | Basic + BMI*sex | 2.79 | 1.97 | 3.60 | <0.001 |
| RVESV | ml | Basic + BMI*sex | 0.87 | 0.39 | 1.36 | <0.001 |
| RVSV | ml | Basic + BMI*sex | 1.92 | 1.40 | 2.43 | <0.001 |
| RVEF | % | Basic + BMI*sex | -0.06 | -0.25 | 0.12 | 0.50 |
| BMI as continuous variable | | | | | | |
| RVEDV | ml | Basic + BMI*sex | 1.42 | 0.81 | 2.02 | <0.001 |
| RVESV | ml | Basic + BMI*sex | 0.19 | -0.17 | 0.56 | 0.29 |
| RVSV | ml | Basic + BMI*sex | 1.22 | 0.84 | 1.61 | <0.001 |
| RVEF | % | Basic + BMI*sex | 0.02 | -0.12 | 0.16 | 0.80 |

* Basic model was adjusted for age, sex, height, height², imaging centre, triglyceride, low density lipoprotein cholesterol, high density lipoprotein cholesterol, glucose, glycated haemoglobin, history of diabetes, history of atrial fibrillation, history of arterial hypertension, and use of lipid-lowering drugs. In addition, the interaction term (BMI*sex) was included. As female sex was coded 1, a positive estimate suggests that BMI has a stronger effect on the RV phenotype in women compared men.

RV end-diastolic volume (RVEDV). RV ejection fraction (RVEF). RV end-systolic volume (RVESV). RV stroke volume (RVSV).

**Supplementary table 2**– Adjusted least square mean values of right ventricular (RV) imaging phenotypes within each of 3 bands of body mass index (BMI) in the UK Biobank cohort, separately in Women and Men.

| RV phenotype | Unit | Model* | BMI groups | | | p-value† |
| --- | --- | --- | --- | --- | --- | --- |
|  |  |  | Normal weight | Overweight | Obese |  |
|  |  |  | BMI <25 kg/m² | BMI 25-29.9 kg/m² | BMI ≥30 kg/m² |  |
| Women | | | | | | |
| RVEDV | ml | Basic | 126.0 | 133.8 | 144.8 | < 0.001 |
| RVESV | ml | Basic | 53.1 | 56.6 | 61.5 | < 0.001 |
| RVSV | ml | Basic | 72.8 | 77.3 | 83.2 | < 0.001 |
| RVEF | % | Basic | 58.0 | 57.8 | 57.4 | < 0.001 |
| Men | | | | | | |
| RVEDV | ml | Basic | 169.7 | 176.8 | 184.3 | < 0.001 |
| RVESV | ml | Basic | 80.8 | 84.5 | 88.1 | < 0.001 |
| RVSV | ml | Basic | 88.8 | 92.3 | 96.2 | < 0.001 |
| RVEF | % | Basic | 52.5 | 52.3 | 52.1 | < 0.001 |

* Basic model was adjusted for age, sex, height, height², imaging centre, triglyceride, low density lipoprotein cholesterol, high density lipoprotein cholesterol, glucose, glycated haemoglobin, history of diabetes, history of atrial fibrillation, history of arterial hypertension, and use of lipid-lowering drugs.

^†^ P-values were generated from linear regression models with BMI bands treated as an ordinal variable.

RV end-diastolic volume (RVEDV). RV ejection fraction (RVEF). RV end-systolic volume (RVESV). RV stroke volume (RVSV).

**Supplementary table 3** –Variants included in genetic instrument on body mass index (BMI).

|  | UK Biobank imaging cohort | | | | Weights on BMI from Locke et al. | |
| --- | --- | --- | --- | --- | --- | --- |
| RSID | REF | ALT | MAF | Missing | Beta | P |
| rs1000940 | A | G | 0.302 | 0.002 | 0.0192 | 1.284E-08 |
| rs10132280 | C | A | 0.300 | 0.019 | -0.023 | 1.141E-11 |
| rs1016287 | C | T | 0.299 | 0.002 | 0.0229 | 2.253E-11 |
| rs10176391 | C | G | 0.010 | 0.007 | -0.0793 | 2.635E-09 |
| rs10182181 | A | G | 0.486 | 0.007 | 0.0307 | 8.777E-24 |
| rs10938397 | A | G | 0.434 | 0.000 | 0.0402 | 3.205E-38 |
| rs10968576 | A | G | 0.324 | 0.000 | 0.0249 | 6.607E-14 |
| rs11030104 | A | G | 0.202 | 0.002 | -0.0414 | 5.557E-28 |
| rs11030107 | A | G | 0.264 | 0.001 | 0.0297 | 3.146E-17 |
| rs11057405 | G | A | 0.105 | 0.000 | -0.0307 | 2.019E-08 |
| rs11074422 | A | T | 0.336 | 0.015 | 0.0188 | 3.337E-08 |
| rs11074446 | T | C | 0.127 | 0.024 | -0.0256 | 1.306E-08 |
| rs11075986 | C | G | 0.078 | 0.002 | -0.0423 | 1.234E-12 |
| rs11126666 | G | A | 0.255 | 0.005 | 0.0207 | 1.332E-09 |
| rs11165643 | T | C | 0.407 | 0.004 | -0.0218 | 2.07E-12 |
| rs11191560 | T | C | 0.077 | 0.000 | 0.0308 | 8.446E-09 |
| rs11583200 | T | C | 0.387 | 0.012 | 0.0177 | 1.479E-08 |
| rs11625769 | G | C | 0.336 | 0.020 | 0.019 | 1.701E-08 |
| rs1167827 | G | A | 0.439 | 0.000 | -0.0202 | 6.333E-10 |
| rs11688816 | G | A | 0.457 | 0.012 | -0.0172 | 1.893E-08 |
| rs11727676 | T | C | 0.095 | 0.000 | -0.0358 | 2.55E-08 |
| rs11847697 | C | T | 0.044 | 0.000 | 0.0492 | 3.99E-09 |
| rs12286929 | G | A | 0.474 | 0.002 | -0.0217 | 1.31E-12 |
| rs12401738 | G | A | 0.380 | 0.007 | 0.0211 | 1.145E-10 |
| rs12429545 | G | A | 0.129 | 0.017 | 0.0334 | 1.094E-12 |
| rs12446632 | G | A | 0.143 | 0.000 | -0.0403 | 1.477E-18 |
| rs12566985 | A | G | 0.438 | 0.002 | 0.0242 | 3.282E-15 |
| rs12885454 | C | A | 0.357 | 0.003 | -0.0207 | 1.943E-10 |
| rs12940622 | G | A | 0.441 | 0.002 | -0.0182 | 2.494E-09 |
| rs12962523 | C | T | 0.207 | 0.010 | -0.0342 | 2.002E-11 |
| rs12996547 | C | T | 0.344 | 0.023 | 0.0246 | 3.667E-14 |
| rs12999373 | G | A | 0.261 | 0.000 | -0.0199 | 4.1E-08 |
| rs13021737 | G | A | 0.172 | 0.000 | -0.0601 | 1.113E-50 |
| rs13078960 | T | G | 0.200 | 0.008 | 0.0297 | 1.737E-14 |
| rs13107325 | C | T | 0.072 | 0.000 | 0.0477 | 1.825E-12 |
| rs1317006 | T | C | 0.297 | 0.003 | 0.0214 | 4.968E-10 |
| rs13191362 | A | G | 0.125 | 0.004 | -0.0277 | 7.339E-09 |
| rs1375561 | T | C | 0.345 | 0.028 | -0.0179 | 9.927E-09 |
| rs1477199 | A | G | 0.134 | 0.002 | 0.0242 | 4.558E-08 |
| rs1516725 | C | T | 0.137 | 0.004 | -0.0451 | 1.886E-22 |
| rs1528435 | T | C | 0.383 | 0.002 | -0.0178 | 1.196E-08 |
| rs1558902 | T | A | 0.399 | 0.000 | 0.0818 | 7.51E-153 |
| rs16851483 | G | T | 0.066 | 0.000 | 0.0483 | 3.548E-10 |
| rs16951275 | T | C | 0.226 | 0.000 | -0.0311 | 1.911E-17 |
| rs17001654 | C | G | 0.144 | 0.026 | 0.0306 | 7.76E-09 |
| rs17024393 | T | C | 0.025 | 0.002 | 0.0658 | 7.029E-14 |
| rs17066842 | G | A | 0.039 | 0.002 | -0.0626 | 6.403E-14 |
| rs17094222 | T | C | 0.212 | 0.007 | 0.0249 | 5.942E-11 |
| rs17405819 | T | C | 0.297 | 0.000 | -0.0224 | 2.07E-11 |
| rs17724992 | A | G | 0.269 | 0.011 | -0.0194 | 3.415E-08 |
| rs1808579 | C | T | 0.488 | 0.002 | -0.0167 | 4.169E-08 |
| rs1928295 | T | C | 0.428 | 0.000 | -0.0188 | 7.91E-10 |
| rs1942873 | C | G | 0.207 | 0.000 | -0.0248 | 2.916E-09 |
| rs2033529 | A | G | 0.286 | 0.007 | 0.019 | 1.388E-08 |
| rs2033732 | C | T | 0.255 | 0.000 | -0.0192 | 4.889E-08 |
| rs205262 | A | G | 0.265 | 0.002 | 0.0221 | 1.753E-10 |
| rs2075650 | A | G | 0.145 | 0.000 | -0.0258 | 1.247E-08 |
| rs2112347 | T | G | 0.358 | 0.000 | -0.0261 | 6.191E-17 |
| rs2121279 | C | T | 0.127 | 0.006 | 0.0245 | 2.313E-08 |
| rs2176598 | C | T | 0.245 | 0.000 | 0.0198 | 2.971E-08 |
| rs2207139 | A | G | 0.168 | 0.000 | 0.0447 | 4.126E-29 |
| rs2245368 | T | C | 0.164 | 0.000 | 0.0317 | 3.187E-08 |
| rs2287019 | C | T | 0.179 | 0.018 | -0.036 | 4.585E-18 |
| rs2365389 | C | T | 0.410 | 0.009 | -0.02 | 1.629E-10 |
| rs2650492 | G | A | 0.294 | 0.021 | 0.0207 | 1.915E-09 |
| rs2817419 | A | G | 0.266 | 0.009 | -0.0275 | 3.662E-15 |
| rs2820292 | C | A | 0.435 | 0.000 | -0.0195 | 1.834E-10 |
| rs29941 | G | A | 0.326 | 0.000 | -0.0182 | 2.407E-08 |
| rs3101336 | C | T | 0.403 | 0.000 | -0.0334 | 2.661E-26 |
| rs3736485 | G | A | 0.460 | 0.010 | 0.0176 | 7.412E-09 |
| rs3810291 | A | G | 0.325 | 0.000 | -0.0283 | 4.812E-15 |
| rs3817334 | C | T | 0.408 | 0.000 | 0.0262 | 5.145E-17 |
| rs3849570 | C | A | 0.347 | 0.000 | 0.0188 | 2.601E-08 |
| rs3888190 | C | A | 0.398 | 0.000 | 0.0309 | 3.14E-23 |
| rs4256980 | G | C | 0.347 | 0.006 | -0.0209 | 2.9E-11 |
| rs4280233 | G | T | 0.054 | 0.009 | -0.0374 | 3.001E-08 |
| rs4671328 | G | T | 0.449 | 0.028 | 0.0215 | 6.217E-09 |
| rs4740619 | T | C | 0.450 | 0.002 | -0.0179 | 4.564E-09 |
| rs543874 | A | G | 0.206 | 0.000 | 0.0482 | 2.618E-35 |
| rs6477694 | T | C | 0.354 | 0.015 | 0.0174 | 2.673E-08 |
| rs6499653 | C | T | 0.241 | 0.036 | 0.0269 | 2.321E-13 |
| rs6567160 | T | C | 0.232 | 0.001 | 0.0556 | 3.93E-53 |
| rs657452 | G | A | 0.391 | 0.015 | 0.0227 | 5.482E-13 |
| rs6804842 | G | A | 0.427 | 0.013 | -0.0185 | 2.476E-09 |
| rs7138803 | G | A | 0.365 | 0.000 | 0.0315 | 8.153E-24 |
| rs7141420 | T | C | 0.483 | 0.025 | -0.0235 | 1.23E-14 |
| rs7203521 | A | G | 0.390 | 0.001 | -0.0326 | 3.455E-24 |
| rs7243357 | T | G | 0.176 | 0.008 | -0.0217 | 3.857E-08 |
| rs758747 | C | T | 0.274 | 0.028 | 0.0225 | 7.473E-10 |
| rs7599312 | G | A | 0.267 | 0.031 | -0.022 | 1.173E-10 |
| rs7899106 | A | G | 0.049 | 0.003 | 0.0395 | 2.96E-08 |
| rs7903146 | C | T | 0.290 | 0.000 | -0.0234 | 1.112E-11 |
| rs879620 | T | C | 0.385 | 0.008 | -0.0244 | 1.061E-09 |
| rs9400239 | C | T | 0.297 | 0.007 | -0.0188 | 1.613E-08 |
| rs9579083 | G | C | 0.183 | 0.004 | 0.0295 | 3.461E-10 |
| rs9675886 | A | G | 0.281 | 0.000 | 0.0349 | 6.924E-25 |
| rs9925964 | A | G | 0.357 | 0.004 | -0.0192 | 8.108E-10 |
| rs9945063 | C | T | 0.238 | 0.000 | 0.0217 | 1.353E-08 |

Weights and levels of significance were obtained from summary-level statistics provided by the GIANT consortium meta-analysis on body mass index (BMI), published by Locke et al. (2015). The summary data were downloaded from https://portals.broadinstitute.org/collaboration/giant/index.php/GIANT_consortium_data_files on August 11, 2024. Variants were clumped for linkage disequilibrium in the UK Biobank imaging cohort with r² < 0.1 within a window of ±500 kb, using PLINK 2.0. Weights are aligned to alternative allele (ALT), the minor allele.

Minor allele frequency (MAF). Reference allele (REF). Reference single nucleotide polymorphism identity (RSID).

**Supplementary table 4** – Results of a two-stage least squares regression model (one-sample Mendelian randomization) that includes an interaction term between BMI and sex to assess the effect of BMI on right ventricular (RV) imaging phenotypes.

| RV phenotype |  | Model* | Estimate | Standard error | p-value |
| --- | --- | --- | --- | --- | --- |
| RVEDV | ml | Basic + BMI*sex | -0.5 | 0.6 | 0.45 |
| RVESV | ml | Basic + BMI*sex | -0.3 | 0.4 | 0.40 |
| RVSV | ml | Basic + BMI*sex | -0.2 | 0.4 | 0.67 |
| RVEF | % | Basic + BMI*sex | 0.0 | 0.1 | 0.87 |

* Basic adjustment included age, sex, height, height², genotype array, assessment centre, and the first 10 genetic principal components. In addition, the interaction term between observed or genetically predicted BMI and sex was included in both stages.

RV end-diastolic volume (RVEDV). RV ejection fraction (RVEF). RV end-systolic volume (RVESV). RV stroke volume (RVSV).

**Supplementary Table 5** – Results from MR-Egger regression intercept test assessing directional pleiotropy, i.e., whether the genetic variants used as instrumental variables affect the outcome through pathways other than the exposure.

| RV phenotype | Egger intercept | SE | p-value |
| --- | --- | --- | --- |
| RVEDV | -0.00035 | 0.00205 | 0.86 |
| RVESV | -0.00147 | 0.00224 | 0.51 |
| RVSV | 0.00069 | 0.00210 | 0.74 |
| RVEF | 0.00248 | 0.00270 | 0.36 |

RV end-diastolic volume (RVEDV). RV ejection fraction (RVEF). RV end-systolic volume (RVESV). RV stroke volume (RVSV). Standard error (SE).

**Supplementary Table 6** – Results from outlier-excluded Mendelian randomisation using the inverse variance–weighted (IVW) method. MR-PRESSO was used to identify horizontal pleiotropic outliers in the analyses of RVEDV and RVESV (rs11126666 in both analyses).

| RV phenotype | Instrument variable | Estimate | Standard error | p-value |
| --- | --- | --- | --- | --- |
| RVEDV | All variants | 0.16 | 0.03 | 1.68E-09 |
|  | Outlier excluded | 0.16 | 0.03 | 7.68E-10 |
| RVESV | All variants | 0.14 | 0.03 | 1.44E-06 |
|  | Outlier excluded | 0.14 | 0.03 | 1.16E-06 |

RV end-diastolic volume (RVEDV). RV ejection fraction (RVEF). RV end-systolic volume (RVESV). RV stroke volume (RVSV).

**Supplementary Table 7** - Results of a two-sample Mendelian randomization of genetically predicted BMI (exposure) and on right ventricular (RV) imaging phenotypes (outcome). Summary-level data from genome-wide association on the UK Biobank imaging cohort were obtained from different sets of data, two sex-combined and one sex-stratified.

| RV phenotype | Method | PMID: 35697868 | | PMID: 35697867 | | PMID: 39374572 | | | | |
| --- | --- | --- | --- | --- | --- | --- | --- | --- | --- | --- |
|  |  |  |  |  |  | Women | | Men | | p-value for sex-related heterogeneity |
|  |  | Estimate (95% CI) | p-value | Estimate (95% CI) | p-value | Estimate (95% CI) | p-value | Estimate (95% CI) | p-value |  |
| RVEDV | IVW | 0.2 (0.1 - 0.3) | <0.001 | 0.3 (0.2 - 0.4) | <0.001 | 0.2 (0.1 - 0.3) | <0.001 | 0.2 (0.2 - 0.3) | <0.001 | 0.771 |
|  | MR-Egger | 0.3 (0.1 - 0.5) | 0.015 | 0.4 (0.2 - 0.5) | <0.001 | 0.2 (0 - 0.4) | 0.116 | 0.3 (0 - 0.5) | 0.022 | 0.474 |
|  | Weighted median | 0.2 (0.1 - 0.3) | <0.001 | 0.3 (0.2 - 0.4) | <0.001 | 0.2 (0 - 0.3) | 0.019 | 0.2 (0.1 - 0.4) | 0.001 | 0.438 |
|  | Weighted mode | 0.2 (0.1 - 0.4) | 0.004 | 0.3 (0.2 - 0.4) | <0.001 | 0.2 (0 - 0.4) | 0.020 | 0.2 (0 - 0.4) | 0.041 | 0.799 |
| RVESV | IVW | 0.2 (0.1 - 0.3) | <0.001 | 0.3 (0.2 - 0.3) | <0.001 | 0.2 (0.1 - 0.3) | <0.001 | 0.2 (0.1 - 0.3) | <0.001 | 0.778 |
|  | MR-Egger | 0.3 (0.1 - 0.5) | 0.014 | 0.3 (0.1 - 0.5) | <0.001 | 0.3 (0 - 0.5) | 0.045 | 0.2 (0 - 0.4) | 0.055 | 0.811 |
|  | Weighted median | 0.2 (0.1 - 0.3) | <0.001 | 0.3 (0.2 - 0.3) | <0.001 | 0.2 (0.1 - 0.3) | 0.002 | 0.2 (0.1 - 0.4) | 0.001 | 0.714 |
|  | Weighted mode | 0.2 (0.1 - 0.4) | 0.004 | 0.3 (0.2 - 0.5) | <0.001 | 0.2 (0.1 - 0.4) | 0.010 | 0.2 (0 - 0.4) | 0.033 | 0.386 |
| RVSV | IVW | 0.2 (0.1 - 0.3) | <0.001 | 0.3 (0.2 - 0.3) | <0.001 | 0.2 (0.1 - 0.2) | <0.001 | 0.2 (0.1 - 0.3) | <0.001 | 0.694 |
|  | MR-Egger | 0.2 (0 - 0.3) | 0.083 | 0.4 (0.2 - 0.5) | <0.001 | 0.1 (-0.1 - 0.3) | 0.513 | 0.2 (0 - 0.4) | 0.082 | 0.452 |
|  | Weighted median | 0.1 (0 - 0.2) | 0.022 | 0.3 (0.2 - 0.3) | <0.001 | 0.1 (-0.1 - 0.2) | 0.292 | 0.1 (0 - 0.3) | 0.048 | 0.314 |
|  | Weighted mode | 0.1 (0 - 0.3) | 0.109 | 0.3 (0.2 - 0.4) | <0.001 | 0 (-0.1 - 0.2) | 0.728 | 0.1 (-0.1 - 0.3) | 0.227 | 0.492 |
| RVEF | IVW | 0 (-0.1 - 0) | 0.378 | -0.1 (-0.1 - 0) | 0.015 | -0.1 (-0.2 - 0) | 0.185 | 0 (-0.1 - 0.1) | 0.509 | 0.839 |
|  | MR-Egger | -0.2 (-0.4 - 0) | 0.108 | 0 (-0.2 - 0.1) | 0.558 | -0.2 (-0.5 - 0) | 0.096 | 0 (-0.3 - 0.2) | 0.796 | 0.934 |
|  | Weighted median | -0.1 (-0.2 - 0) | 0.141 | -0.1 (-0.2 - 0) | 0.074 | -0.1 (-0.2 - 0) | 0.158 | -0.1 (-0.2 - 0) | 0.197 | 0.631 |
|  | Weighted mode | -0.2 (-0.3 - 0) | 0.089 | -0.1 (-0.2 - 0) | 0.102 | -0.1 (-0.3 - 0.1) | 0.195 | -0.1 (-0.3 - 0.1) | 0.294 | 0.311 |

* To test for heterogeneity between the sexes, we used a meta-analysis framework to obtain p-values from Cochran’s Q test for heterogeneity.

Confidence interval (CI). Inverse variance-weighted (IVW). Identifier number used in US-National Library of Medicine PubMed (PMID). RV end-diastolic volume (RVEDV). RV ejection fraction (RVEF). RV end-systolic volume (RVESV). RV stroke volume (RVSV).


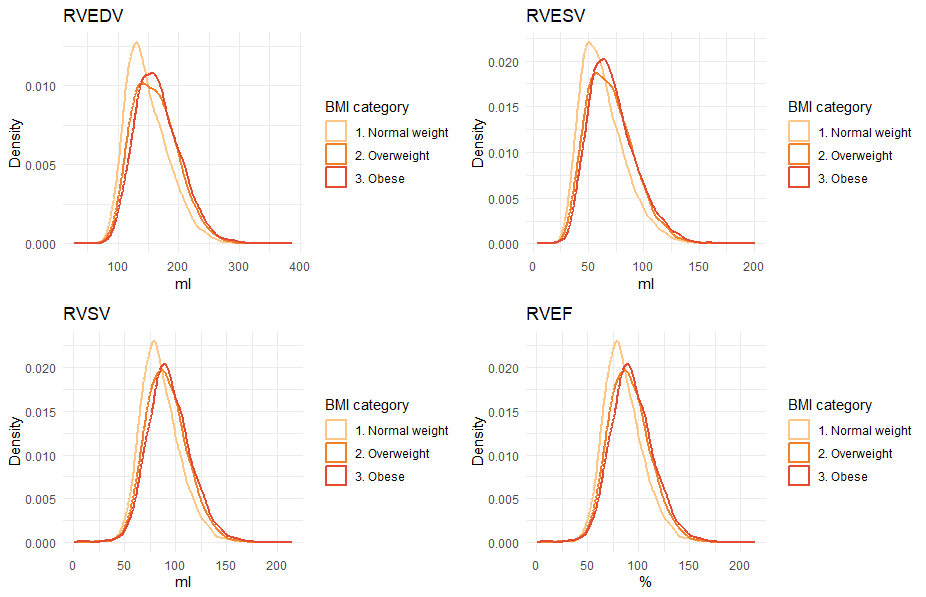


**Supplementary Figure 1** – Distributions of measured right ventricular volumes and ejection fraction according to different categories of body mass index (BMI). Participants were categorized into normal weight (BMI <25 kg/m²), overweight (BMI 25–29.9 kg/m²), and obese (BMI ≥ 30 kg/m²).

RV end-diastolic volume (RVEDV). RV ejection fraction (RVEF). RV end-systolic volume (RVESV). RV stroke volume (RVSV).


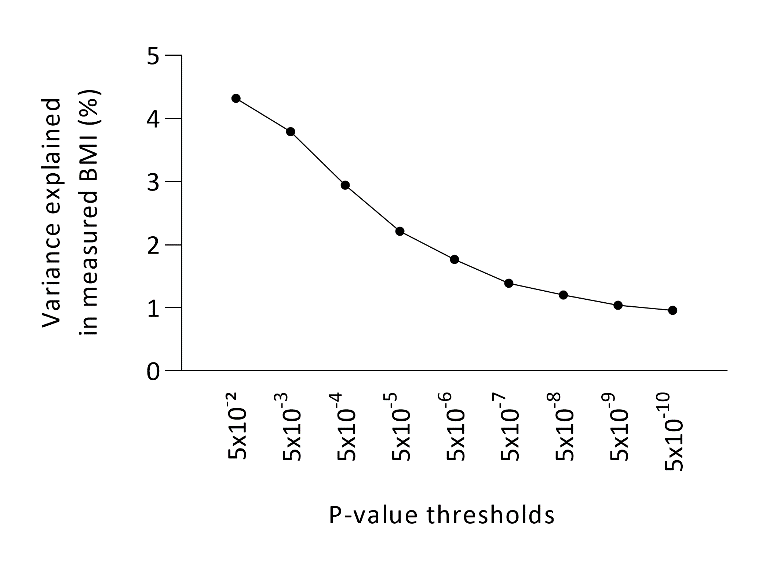


**Supplementary Figure 2**– The variance explained in measured body mass index (BMI) by genetic instruments generated from GIANT summary statistics using different significance threshold for variant selection, followed by clumping in the UK Biobank imaging cohort (r^2^<0.1).


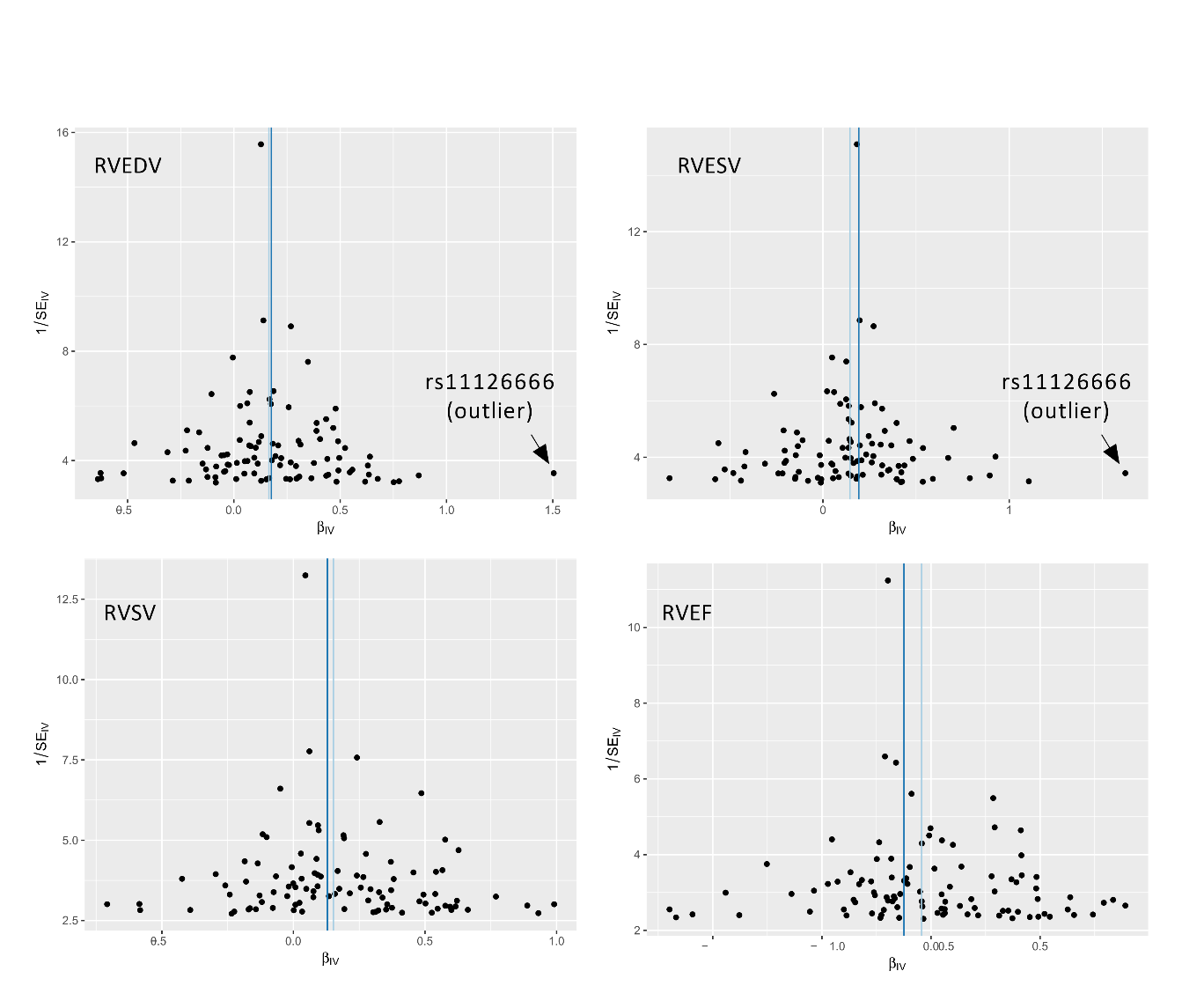


**Supplementary Figure 3** - Funnel plots compare the effect estimates (β) on the outcome with the inverse of the standard error (SE) of each genetic variant within the instrumental variable. Variants with higher precision (lower standard errors) are plotted further up the graphs. Central effect estimates are shown as horizontal lines (light blue for the IVW method and dark blue for MR-Egger). Symmetry around the central estimate suggests the absence of systematic bias, such as directional pleiotropy. MR-PRESSO was used to identify horizontal pleiotropic outliers in the analyses on RVEDV and ESV (rs11126666).

Inverse variance–weighted (IVW). RV end-diastolic volume (RVEDV). RV ejection fraction (RVEF). RV end-systolic volume (RVESV). RV stroke volume (RVSV).


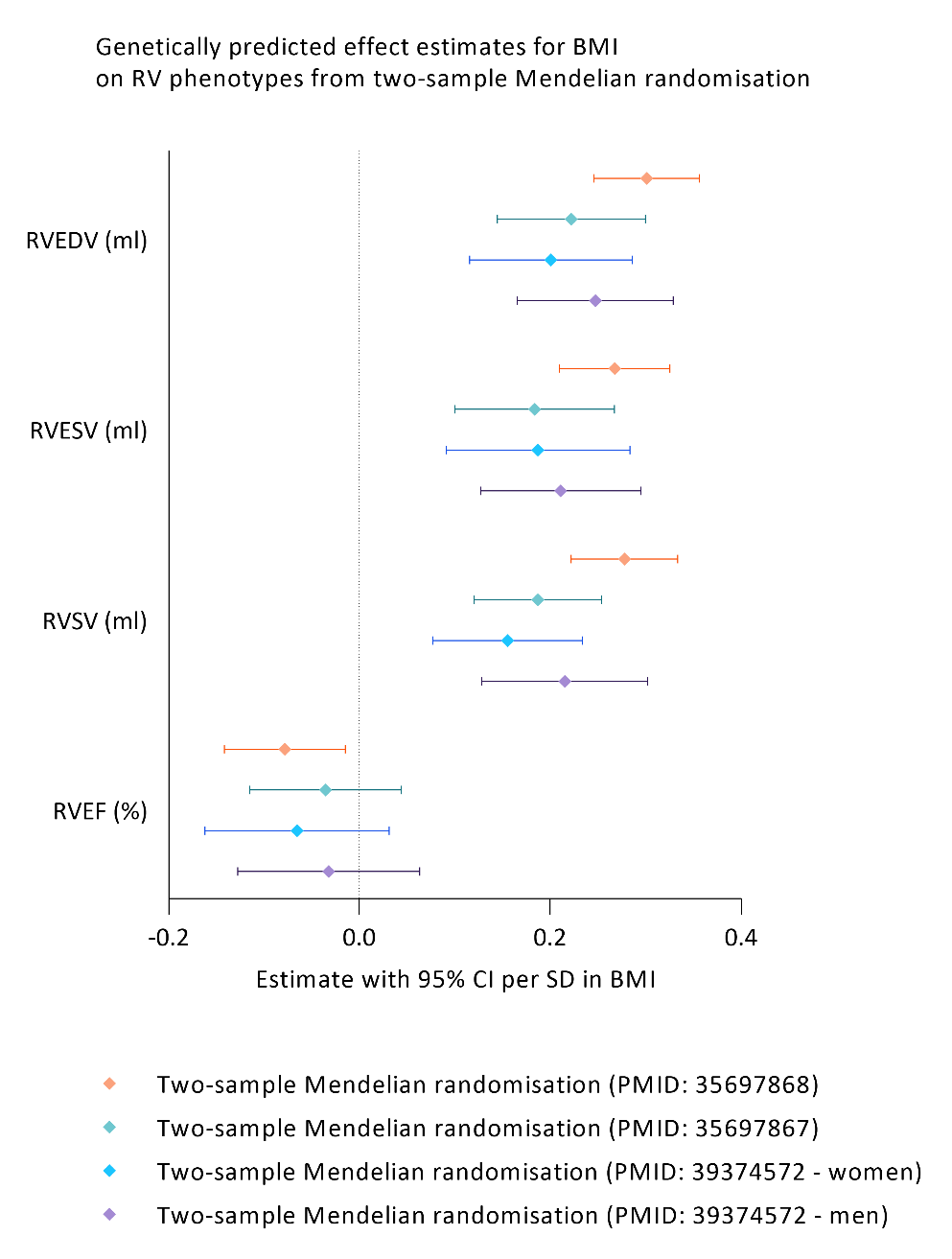


**Supplementary Figure 4** - Results from two-sample Mendelian randomisation studies of genetically predicted BMI (exposure) and on right ventricular (RV) imaging phenotypes (outcome). Summary-level data from genome-wide association on the UK Biobank imaging cohort were obtained from three different sets of data, two sex-combined and one sex-stratified.

Identifier number used in US-National Library of Medicine PubMed (PMID). RV end-diastolic volume (RVEDV). RV ejection fraction (RVEF). RV end-systolic volume (RVESV). RV stroke volume (RVSV). Standard error (SE).
